# Correction to “Revisiting the effectiveness of cognitive‐behavioural therapy for reducing reoffending in the criminal justice system: A systematic review”

**DOI:** 10.1002/cl2.1438

**Published:** 2024-09-02

Smith, A., Roberts, A., Krzemieniewska‐Nandwani, K., Eggins, E., Cook, W., Fox, C., Maruna, S., Wallace, S., & Szifris, K. (2024). Revisiting the effectiveness of cognitive‐behavioural therapy for reducing reoffending in the criminal justice system: A systematic review. *Campbell Systematic Reviews*, *20*, e1425. https://doi.org/10.1002/cl2.142

There is an omission of the word “PROTOCOL” at the beginning of the article title and the correct article title should read as:

PROTOCOL: Revisiting the effectiveness of cognitive‐behavioural therapy for reducing reoffending in the criminal justice system: A systematic review.

The correct article category is PROTOCOL.

The article title and the category have been corrected in the original publication. We apologize for this error.

